# Everybody's Page

**Published:** 1892-08-13

**Authors:** 


### EVERYBODY'S PAGE.

Of the numerous new remedies for all our ills too much is invariably claimed by the over-enthusiastic. Icthyol is no exception. It does not accomplish all it was asserted it would do in eczema, though it appears to have undoubted value as an antiseptic in various affections of the skin, and it is a useful application for erysipelas.

Canada is still in want of emigrants of the right sort. Farm labourers and domestic servants are badly needed, and capable hands are sure of earning a good wage. With the superabundant labour over here this ought to be remedied with little difficulty, and with much advantage to many hundreds of people. In some districts of Canada, owing to the great scarcity of men, it was found almost impossible last season to stack the crops before winter came on.

If credit were always given where credit is due we should have to acknowledge that many scientific improvements are due, indirectly at least, to the wicked ways of our little street arabs. The automatic stamp distributors, a few of which have been in use during the past twelve months, and which are likely to prove a great boon to the public, have now been brought to a state of perfection, and will shortly be seen everywhere. The need for various alterations in their working has been demonstrated in a very practical manner by naughty little boys, who show an ingenuity which, if turned to better account, might stand them in good stead later in life. Here is another instance of gratitude being due where cuffs are so often administered instead, for much of the wisdom of the world is gained by careful observation of the habits of small boys.

The recent alleged poisoning cases in London have called forth some correspondence on the relative values of various methods of disposing of the dead. The advocates of the earth to earth and other systems than cremation have trotted out their usual assertions as to the dangers likely to arise if the latter is generally adopted, but argument has been conspicuous by its absence. On the other hand, it has been shown conclusively by Sir Spencer Wells, that the regulations to be complied with before a body is cremated are so stringent, that it is utterly improbable that the necessary certificate would have been forthcoming before cremation could have taken place. There is absolutely nothing in the ordinary mode of burial to deter a would-be poisoner, but cremation can be and is hedged in with precautions which are likely to bring a criminal to justice with much greater speed and certainty than is now the case.

Shall we ever be able to communicate with Mars? Mr. Francis Gatton thinks the possibility is not so remote. His suggestion of signalling by means of mirrors has the merits of simplicity, but to be effective it not only requires the presence of human beings on that planet, but the power of seeing Mars during the daytime.

Trepanning is now often used in the treatment of epileptics. It is stated that this operation has been performed recently in Vienna on persons suffering from the hereditary form of this disease, but the advocates of this method of treatment are sufficiently wise not to claim success till they have had sufficient time to observe the result of their operations. Inasmuch as epilepsy often recurs after disappearing for some time, conclusive judgment on any new method of treatment cannot be given till after the lapse of several years.

The surgeons of the shipping companies which bring immigrants to the United States do not possess the confidence of the inhabitants of the Great Republic. The *New York Medical Record* holds views of them the reverse of complimentary. The danger of imported epidemics cannot, of course, be averted by the utmost vigilance of the quarantine and port health authorities, unless their efforts are supported by competent surgeons. It appears that a short while ago a health officer of the port of New York detected a case of typhus fever in a Russian Jewess, which the ship's surgeon had diagnosed as measles, a significant instance of how diseases can be spread in spite of all precautions.

The brutal onslaught on the ladies near Bickley by an epileptic brings before the public, in a very unpleasant manner, the question of criminality of the insane. Where should the line be drawn between crime and insanity? It may be said that every criminal is insane, but there is a class of professed criminal who cannot urge the plea of irresponsibility, though no doubt this plea is urged in some cases successfully where many people think such success should be impossible. An examination of criminals would no doubt show that a large proportion of them are the offspring of drunkards or epileptics, with a predisposition to crime. Where in such cases is the plea of irresponsibility to be urged? In connection with crime a curious fact has been observed by a French savant, that with every phase in the progress of civilization the prevailing types of crime have varied. This, of course, can readily be accounted for. The criminal, like the drunkard, requires his opportunities. Piracy and highway robbery have given way to theft of a less romantic order and stealthy forms of murder.

